# The Value of Liver Transplantation for Methylmalonic Acidemia

**DOI:** 10.3389/fped.2019.00087

**Published:** 2019-03-21

**Authors:** Yi-Zhou Jiang, Li-Ying Sun

**Affiliations:** ^1^Intensive Care Unit, Beijing Friendship Hospital, Capital Medical University, Beijing, China; ^2^Liver Transplantation Center, Clinical Center for Pediatric Liver Transplantation, National Clinical Research Center for Digestive Diseases, Beijing Friendship Hospital, Capital Medical University, Beijing, China; ^3^Beijing Key Laboratory of Tolerance Induction and Organ Protection in Transplantation, Beijing, China

**Keywords:** methylmalonic acidemia, methylmalonic acid, liver transplantation, metabolic, decompensation

## Abstract

**Introduction:** MMA is a rare autosomal recessive disorder with the manifestation of recurrent and severe episodes of acute metabolic decompensation or a variety of long-term complications that require timely treatment. While conventional long-term medical and dietary management cannot prevent rapid progression of conditions in patients with severe complications, LT, or CKLT has become an option.

**Methods:** We reviewed the literature for MMA patients undergoing LT/CKLT published since 2006, and data on metabolic decompensation status, protein dietary, neurological damage, renal insufficiency, and developmental delay before and after transplantations were compared to evaluate the clinical value of the procedure in the treatment of MMA.

**Results:** To date, some successful LTs/CKLT procedures have prolonged survival and resulted in better quality of life in patients (lowered urine/plasma MMA levels but still much higher than normal, reduced onset of metabolic stroke, occasional improved developmental delay, and relaxed protein diet), although these procedures cannot reverse neurological damage or thoroughly stop the progress of complications, such as renal dysfunction.

**Conclusion:** LT is the only effective treatment for MMA patients with recurrent metabolic decompensation. However, it is still possible that neurological and renal damage remains irreversible. Metabolism-correcting medications should be administered even after surgery.

## Introduction

Methylmalonic acidemia (MMA) is a rare genetic metabolic disease, and most of its cases are autosomal recessive. It is estimated that the incidence of MMA in Western populations ranges from 1:48,000 to 1:61,000 births, and the overall incidence of isolated MMA is believed to be ~1:50,000. In some populations across the world, the incidence is much higher (1). A large amount of methylmalonic acid accumulates in patients with MMA due to the deficiency of MCM or the synthesis of its active cofactor, adenosylcobalamin (AdoCbl). Defects in cobalamin (vitamin B12) metabolism may also manifest as homocystinuria (called combined MMA) (2). Most severe, early-onset MMA cases are caused by mutations in the MUT gene located on chromosome 6p21 and are called isolated MMA (3). To date, 272 different MUT mutations have been discovered, most of which are missense or nonsense mutations (4).

MMA is one of the most frequent organic acidurias. Propionic acidemia (PA), another inborn errors of metabolism, has defects in enzyme propionyl-CoA carboxylase (PCC) which also lead to accumulation of toxic metabolites by disrupting the normal amino acid metabolism (5). The severity of illness of patients with isolated MMA varies. For patients with severe cases, the onset of MMA begins in the neonatal or infancy period, with recurrent metabolic decompensation, or metabolic crisis. The manifestations lack specificity in clinical symptoms, but the most common symptoms are vomiting, metabolic acidosis, and high blood ammonia hyperammonemia which is usually associated with decreased levels of glutamine (6). If without prompt identification and appropriate management, one can become progressively encephalopathic, and ultimately progresses to coma and even death. The mortality of mut MMA was nearly 60% or even higher in the 1980s and improved only slightly, to about 40%, by the first decade in the 2000s (7–10). Currently, conventional therapies are symptom-based treatment and dietary and nutritional management. The mainstay of nutrition therapy is low protein intake through limitation of the propionic acid precursor amino acids, isoleucine, valine, methionine, and threonine, to reduce elevated concentrations of metabolites and by supplementing with precursor-free infant amino formula to ensure nutritional requirements are met. Administration of L-carnitine (to help enhance the elimination of propionyl group), sodium bicarbonate, and vitamin B12 is also included (1). However, patients with isolated MMA often have earlier and more severe clinical manifestations, most of which are coupled with long-term complications involving multiple organs and frequent hospitalization due to recurrent metabolic decompensation (11, 12). MMA continues to cause significant morbidity and mortality due to acute and chronic multisystemic damages such as severe metabolic stroke events which may be associated with basal ganglia necrosis. Since prognosis of this disorder is strongly influenced by the time of metabolic decompensation and end-organ injuries, improving metabolic stability, and possibly change the natural history of MMA is significant. Other treatments are urgently needed to improve the long-term survival.

Since most metabolic conversion of MMA occurs in the liver, liver transplant (LT) can help patients regain enzyme activity and improve metabolic capacity (13). Since LT was performed for a patient with MMA for the first time in 1997 (14), LT and combined kidney-liver transplant (CKLT) have been increasingly used to treat MMA cases where frequent metabolic decompensation cannot be relieved by diet therapy and medication and have successfully saved many lives. These results emphasize the urgent need to better understand this kind of therapeutic strategy. In our review, we provide a literature overview of MMA patients undergoing LT or CKLT to explore the value of LT in the treatment of MMA.

## Methods

The literature about MMA patients undergoing LT/CKLT published since 2006 was reviewed in PubMed Database. The key words used include methylmalonic acidemia, methylmalonic acid, MCM deficiency, liver transplantation, and combined liver-kidney transplantation. Age at transplant, procedure and follow-up period were reviewed in the articles. Data on the onset frequency of metabolic decompensation or crisis time, MMA levels in the plasma and urine, protein diet, neurological damage, renal insufficiency, and developmental delay before and after transplantations were analyzed to better evaluate the clinical value of this procedure for MMA. Research of animal model and review article were excluded. Papers with irrelevant content to the topic we focused were also not included.

## Results

We summarized the characteristics of reported patients with MMA undergoing LT/CKLT since 2006 (Table 1). All patients were alive when the literature was published, except for one patient who died of sepsis on postoperative day 44 (15).

**Table 1:** Outcomes of LT/CKLT for patients with MMA.

**References**	**Age at Tx**	**Procedure**	**Follow-up**	**Metabolic decompensation/crisis time**		**MMA level (P/CSF: nmol/mL U:** **μmol/mmol Cr)**		**Dietary protein (g/kg/d)**		**Neurological damage/ DQ**		**Renal dysfunction (eGFR:mL/min/1.73 m**^****2****^		**Developmental delay/SD of height**	
				**Pre**	**Post**	**Pre**	**Post**	**Pre**	**Post**	**Pre**	**Post**	**Pre**	**Post**	**Pre**	**Post**
Kaplan et al. (27)	19 m	OLT	10 y	Y	Y (only twice)	P:574 ± 431	P:220 ± 79	1.7	NA	Increased subarachnoid space	Acute lesion in right globus pallidus, then resolved^&^	N	eGFR = 77	Between the 25th and 50th percentiles	−2SD
						U:9307 ± 4923	U:3656 ± 2271								
						CSF: 1103	CSF:901 ± 263								
Mc Guire et al. (21)	5 y	CKLT (OLT)	10 m	Y	N	P:20–2591	P:25–525	1.95	NA	Y (cerebellar stroke)	Y	Y	N	Failure to thrive	NA
						U:1101–13962	U:116–1895								
Chen et al. (19)	0.9–2.1 y	LDLT (*n* = 4)	0.2–7.7 y	2.73/y	0.08/y	P:87.5–204	P:63.2–87	0.66–1.00	1.37–2.80	NA	NA	N	N	Development all continued	
Morioka et al. (15) Kamei et al. (16)	7–90 m	LDLT (*n* = 7)	19–53 m	Y	N	P:268.0	P:99.4	1.0	3.0	The global cognitive index of the McCarthy scale and the Denver development quotient were improved but did not reach normal values		N	N	−2	−2
						P:47.0	P:59.2	1.2	2.5			N	N	−2	−2
						P:143.0	P:36.4	0.7	2.5			N	N	−3.14	−2
						P:39.0	P:29.3	2.0	3.0			N	N	−2	−1
						P:375.0	P:87.8	1.0	2.5			N	N	−1.3	−0.6
						P:1970.0	P:232.0	–^#^	–			Y	–	–	–
						P:166.0	P:13.8	1.5	2.5			N	N	−3	−2
		LDLT (*n* = 3)				P:278.0	P:59.6	NA	NA	NA	NA	N	N	NA	NA
						P:702.0	P:124.4								
						P:255.0	P:8.5								
Vernon et al. (29)	28 y	CKLT	18 m	Y	N	P: 6965 ± 1638	P:234 ± 100	Restricted	Not restricted	Optic neuropathy, leukoencephalopathy	Stable visual function, tremor persists	Y	N	Worsening generalized debility	Able to walk
Spada et al. (28)	3 y	Whole LT	12 y	Y	N	P: sustained (~80%) and stable reduction	0.8	1.5	Normal intellectual development	N	Y	NA	NA		
	9 m	Split-LT	2 y	Y	N	P:124.4	P:43.5	0.8	1.8	Adequate neurologic development		N	N	NA	NA
Niemi et al. (18)	Mean 8.2 y (0.8–20.7)	LT^*^ (*n* = 6) CKLT (*n* = 8)	Mean 3.25 ± 4.2 y	Y	N	P:1648 ± 1492	P:305 ± 108	1.6 (Natural protein 0.3–1.9)	1.6 (Natural protein 0.6–1.8)	Maintained neurodevelopmental abilities	Y (*n* = 8)	N	Present in 12 patients (86%)	Maintained or improved	
Khanna et al. (24)	28 y	OLT (domino donor)	11 m	Y	N	P:445.9 ± 257.0	P:333.3 ± 117.7	Y	1.0–1.9 (liberalized)	Increasing neurologic disability	NA	>60	51.0 ± 12.1^†^	Altered gait, and slower speech	NA
						U:5277 ± 1968	U:1068 ± 384								
Sakamoto et al. (20)	7 y	LDLT (*n* = 13)	4–16 y (mean 8.1 y)	0	0	P: ~75–240 (mean)	P: ~5–170 (mean)	1.2	Less	41	53	N	N	−2.0	−2.0
	5 y			3	0			0.7	Less	43	48	N	N	−3.1	−2.0
	1 y			3	0			1.5	1.65–1.8	49	54	N	N	−3.0	−2.0
	8 m			1	0			1.2	1.3	NA	32	N	N	−2.8	−0.2
	11 m			3	1			0.9	1.5	55	48	N	N	−1.4	−1.8
	5 y			5	5			1.7	0.95	NA	23	N	N	−4.3	−4.4
	10 m			2	0			1.5	1.0–1.5	63	55	N	N	−2.5	−1.3
	12 m			2	0			0.7	1.0–1.5	57	42	N	N	−2.5	−1.7
	9 m			3	2			1.3	1.0	NA	NA	N	N	−3.2	−0.6
	8 m			1	0			1.3	1.2	NA	NA	N	N	1.5	0.8
	2 y			3	0			1.0	1.0–1.5	60	54	Y	Y^※^	−3.6	−1.9
	2 y			5	1			2.0	1.0–1.5	NA	NA	N	N	−3.6	−3.2
	10 m			1	0			1.5	Not restricted	70	NA	N	N	−0.7	0.0
Critelli et al. (23)	6.6 y	Kidney/split liver	3.1 y	Y	N	P: 745 (mean)	P: 154.9 (mean)	1.6–2.0	1	NA	NA	56	78	Mild	NA
	21.6 y	CKLT	1.6 y	Y	N			1.45–1.75	1.0–1.1			40	70	Extremely low to borderline	
	7.4 y	CKLT	4.1 y	Y	N			1.6–2.0	1.43			66.2	142	Moderate to severe	
	15.5 y	CKLT	11.6 y	Y	N			1.3	0.76–0.95			40	68^§^	Mild	
	9.4 y	CKLT	3.6 y	Y	N			0.98–1.18	1.3–1.5			65	88	No formal testing	
	1.9 y	OLT	1 y	Y	N			0.83	1.0–1.2			96.8	128	Borderline	

*NA, not available; OLT, orthotopic liver transplantation; LDLT, living donor liver transplantation*.

& *72 days post-transplantation, MRI with diffusion-weighted imaging of brain demonstrated an acute lesion in the right globus pallidus but has never manifested clinical signs of extrapyramidal tract disease. Subsequent MRI 18 months later showed resolution of the basal ganglion lesion*.

# *Died of sepsis on postoperative day 44*.

* *One underwent liver retransplantation because of hepatic artery thrombosis*.

† *The postoperative period was complicated by acute kidney injury. The renal function improved progressively*.

※ *Acute renal failure occurred after using contrast medium for endoscopic retrograde cholangiopancreatography*.

§ *Underwent a renal biopsy 17 months after CLKT, which showed mild tubulointerstitial injury*.

### Improvement in Metabolic Decompensation and Protein Diet After LT

Koichi Kamei et al. (16) attempted to reduce MMA levels in plasma by preoperative dialysis to reduce the risk of postoperative metabolic decompensation. The results showed that, although the MMA level was significantly reduced (*n* = 10), different degrees of acidosis still occurred (*n* = 3), and one patient died (*n* = 1). It was obvious that dialysis failed to produce satisfactory results. LT, on the other hand, can quickly relieve the state of metabolic decompensation, and the recurrence rate during follow-up was significantly reduced (17).

At present, it has been confirmed by many reports that metabolic crisis recurrence can be eliminated in patients following transplant and more than 80% no longer have metabolic decompensation and acidosis, and the frequency is reduced in the remaining patients (the follow-up time ranges from 2 m to 16 y) (18–20). The state of lethargy was also completely eliminated according to Morioka et al. (15). Plasma and urine MMA levels steadily decreased to different degrees, and some reached as high as 90%, even with increased protein intake (15, 18–24). However, MMA levels are still more than 100 times or even 1,000 times higher than those of normal people (25) and are concentrated mainly in extrahepatic tissues (26). The concentration of MMA in cerebrospinal fluid (CSF) was not corrected after LT (27). Almost all patients still maintained L-carnitine supplementation to correct metabolic disorders. For the children who suffered neonatal-onset MMA and received early LT (*n* = 2), there was no metabolic decompensation, and the plasma MMA concentration continued to decline steadily after LT. One case was followed up for 12 years (28).

Most patients still maintain a low protein diet after surgery, while more than 70% have increased total protein intake, and two patients even have no restrictions (20, 29). The latest research suggests that acidosis status is significantly improved over the perioperative period by implementing a strategy of carbohydrate minimization with gradual but early lipid and protein introduction (23).

### Improvement in Neurological Damage and Physical Retardation After LT

Due to the accumulation of MMA in the brain and recurrent acidosis, the manifestations of MMA in patients often include physical and neurodevelopmental disorders (30). The reported data showed that ~25–65% of the patients suffered developmental delay, 30–45% suffered motor dysfunction (1), and approximately half of the patients suffered mental retardation (7). Sakamoto et al. (20), reported that the mean developmental quotient (DQ) levels of children were 51 ± 9 and 50 ± 5 (*P* = 0.65) before and after living donor liver transplantation (LDLT), respectively, and no further deterioration occurred after LT (*n* = 13) (20). The patient's nervous system damage was partially ameliorated, even if the acute exacerbation of chronic bilateral optic neuropathy occurred shortly after surgery, and the situation improved later and remained stable (29) (*n* = 1). MRI of the brain of one patient who had never manifested clinical signs of extrapyramidal tract disease demonstrated an acute lesion in the right globus pallidus on postoperative day 72, and subsequent MRI 18 months later showed resolution of the basal ganglion lesion (27). In a special case, acute neurological deterioration occurred in the 5th year after LT, characterized by sudden changes in consciousness, aphasia, and hypotonia. The MRI results showed bilateral basal ganglion symmetry (*n* = 1) (25). Studies have shown that the concentration of MMA levels in CSF may remain high for several months, and even several years, after LT, which can lead to basal ganglia damage, dyskinesia, sensory neurological deafness, and other neurological complications (21, 27, 29). Patients were more likely to have seizure episodes or persistent tremors (29) in the perioperative period, which is probably a response to antibiotics and antifungal therapy after transplantation (24).

Due to short-term follow-up, most articles did not mention improvement in developmental status. For others, their developmental delay was maintained or improved. The height of most patients was significantly improved. The effect was even better when LT was performed within 1 year of age, and physical development could be restored to a normal range within 2.5 years following LT and was similar to the height of normal children of the same age group (*n* = 14) (18). In a study with an average follow-up of 8.1 years, the standard deviation (SD) of height of 77% (10/13) of patients improved, although it did not catch up with the age-matched normal value (20). Some children also showed improved athletic, learning, and social abilities, including normal schooling (18, 25).

### Improvement in Renal Insufficiency After LT

The accumulation of MMA can also cause visible damage to other organs, especially the kidney. By undergoing CKLT, most patients with pre-existing renal dysfunction recovered. However, Sakamoto et al. (20) reported a case with renal dysfunction (creatinine level rising) before LT. The renal function of the patient failed to recover after LT, and acute renal failure occurred after using contrast medium for endoscopic retrograde cholangiopancreatography. Some patients exhibited progressive renal insufficiency even after surgery due to the natural history of the disease or possibly even accelerated by taking immunosuppressive drugs which can cause damage to kidney (24, 28, 31).

In summary, transplantation can prevent patients with MMA from undergoing a metabolic crisis. Urine and plasma MMA levels are significantly reduced, and quality of life is significantly improved after LT. However, it cannot completely cure the disorder. MMA levels are still much higher than normal after LT, and patients still require metabolic-correcting medication as a long-term treatment. In addition, surgery cannot help patients completely avoid renal dysfunction and improve neurological complications (for more details, see Table 1).

## Discussion

Most of the metabolic conversion of propionate occurs in the liver, therefore, LT can help the body regain normal functioning enzymes, thus improving metabolic capacity (32). With sophisticated transplantation techniques and perioperative management experience, most issues related to high mortality caused by surgery have been resolved. At present, organ transplantation has become an effective treatment for isolated MMA.

### For Metabolic Decompensation and Protein Dietary

Metabolic crisis is a fainting episode caused by a sudden worsening of metabolic decompensation for MMA patients (15). According to current reports, compared with traditional diet and medical treatment, LT can quickly alleviate metabolic decompensation of patients with MMA and basically prevent the recurrence of metabolic crisis. For infants with severe MMA, LT is highly recommended (33). However, because the genetic defect of the disease is expressed in multiple organs of the body, the metabolite level is still higher than normal even after LT. The concentrations of MMA in the CFS was not corrected according to the study. It is reasonable to assume that metabolites existing in brain are difficult to transported across the blood brain barrier.

Although dietary restriction cannot totally return to normal, relaxed dietary protein restriction with appropriate amount of natural protein can safely occur in some patients. Some medical professionals believe that because excessive restriction on protein intake may lead to a lack of essential amino acids and cause complications, an appropriate protein diet may be helpful to control MMA (18). Therefore, nutritional management is critical to optimizing the outcomes of MMA patients who are metabolically brittle. Multiple patient- and graft-related factors require us to individualize and adjust medical and nutritional support. A proposed diagnostic and management guideline of MMA/PA was carried out by worldwide experts recently and recommendations about dietary management were included (1). Additional long-term follow-up under close monitoring is required, and the recommended dietary allowance of total protein intake should be practiced.

### For Neurological and Developmental Delay

The widespread distribution of metabolites in the central nervous system has direct neurotoxicity and can induce behavioral abnormalities and seizures in a rat model (22, 34). The basal ganglia of patients with severe MMA are prone to involvement. MRI is generally characterized by demyelination of white matter and abnormal signals in basal ganglia, which were caused by mitochondrial energy metabolism disorders (25). A study found that the basal ganglia have high energy requirements in childhood (35). Therefore, as brain energy metabolism is impaired, the brain is more vulnerable to damage.

Postoperative DQ and other results indicate that slow development still existed and early transplantation was better for children's physical development. One possible reason is that growth retardation is associated with insufficient protein intake and energy, repeated acidosis or vomiting (36). Therefore, early LT is recommended, especially within 1 year of age (28). LT can increase patient tolerance to natural protein, thus increasing the intake of protein in diet, and can provide a stable condition, preventing the onset of acidosis to promote physical development. While some patients with MMA still have persistent damage to the nervous system after surgery. It has been reported that newly synthesized propionyl-CoA in the brain leads to continuous accumulation of MMA, and it is difficult for metabolites already present in the brain to break through the blood-brain barrier and flow out of the CSF (27). Since the MMA levels in the brain after LT may still be high, toxic effects on the brain still exist. Potentially sustained synthesis of MMA in the sac is considered a permanent risk factor for metabolic crisis (22, 27).

### For Renal Damage

A study with a mouse model suggested that mitochondrial dysfunction in the proximal tubules of the kidney is an important pathogenic mechanism of MMA-related renal disease (37). MMA patients with long-term survival may have renal complications due to progressive tubulointerstitial injury (38). It has been reported in the literature that LT should be performed as early as possible to obtain normal renal function (19). However, for patients during the disease progression period, postoperative renal function might continue to deteriorate. For patients with preoperative chronic kidney disease (especially at estimated glomerular filtration rate (eGFR) <20 ml/min/1.73 m^2^), CKLT is effective and recommended (18). While renal function remained unrecoverable or deteriorated in some patients despite a low-protein diet and appropriate medications after transplantation, which may be related to the nephrotoxicity of immunosuppressive agents, such as FK506 (39–41). To date, there are no consensus and guidelines on immunosuppression regimens. But it is reasonable to infer that we can adjust the immunosuppressive regimen such as postponing the administration time for patients with pre-existing renal insufficiency to slow down the progress to renal failure. This protocol has been carried out by our center and the shot-time outcome came out to be a success. Close long-term monitoring of postoperative renal function and strict individualized immunosuppressive therapy are extremely important (42). However, since most MMA patients have low muscle mass and are children, the serum creatinine, and calculated eGFR cannot accurately reflect real renal function (43). A new indicator that can accurately represent renal function of children with MMA needs to be established to avoid kidney transplantation in the future.

## Conclusion

It is important to recognize in our review that LT is a valuable option for MMA patients and has gained some favorable prognosis, though it does not bring about a complete cure. In the future, research on how to reverse neurological damage needs to be conducted to further improve the efficacy of transplantation. Further understanding of the correlation between disease severity, procedure and outcomes will help us determine the individualized type and optimal timing of transplantation.

## Ethics Statement

This article does not contain any studies with human participants or animals performed by any of the authors.

## Author Contributions

Y-ZJ and L-YS contributed to the conception and design of the manuscript. L-YS provided administrative support. Y-ZJ wrote the manuscript. Both authors approved the final version of this manuscript.

### Conflict of Interest Statement

The authors declare that the research was conducted in the absence of any commercial or financial relationships that could be construed as a potential conflict of interest.

## Abbreviations

CLKT: Combined liver-kidney transplantation
CSF: Cerebrospinal fluid
LDLT: Living donor liver transplantation
LT: Liver transplantation
MCM: Methylmalonyl-CoA mutase
MMA: Methylmalonic academia
OLT: Orthotopic liver transplantation.

## References

[1] Baumgartner MR Horster F Dionisi-Vici C Haliloglu G Karall D Chapman KA. Proposed guidelines for the diagnosis and management of methylmalonic and propionic acidemia. Orphanet J Rare Dis. (2014) 9:130. 10.1186/s13023-014-0130-8 25205257 PMC4180313

[2] Fowler B Leonard JV Baumgartner MR. Causes of and diagnostic approach to methylmalonic acidurias. J Inherit Metab Dis. (2008) 31:350–60. 10.1007/s10545-008-0839-4 18563633

[3] Ledley FD Lumetta MR Zoghbi HY VanTuinen P Ledbetter SA Ledbetter DH. Mapping of human methylmalonyl CoA mutase (MUT) locus on chromosome 6. Am J Hum Genet. (1988) 42:839–46. 2897160 PMC1715214

[4] Keyfi F Talebi S Varasteh AR. Methylmalonic acidemia diagnosis by laboratory methods. Rep Biochem Mol Biol. (2016) 5:1–14. 28070528 PMC5214677

[5] Fraser JL Venditti CP. Methylmalonic and propionic acidemias: clinical management update. Curr Opin Pediatr. (2016) 28:682–93. 10.1097/MOP.0000000000000422 27653704 PMC5393914

[6] van der Meer SB Poggi F Spada M Bonnefont JP Ogier H Hubert P Clinical outcome of long-term management of patients with vitamin B12-unresponsive methylmalonic acidemia. J Pediatr. (1994) 125 (6 Pt 1):903–8. 10.1016/S0022-3476(05)82005-0 7996362

[7] Horster F Baumgartner MR Viardot C Suormala T Burgard P Fowler B. Long-term outcome in methylmalonic acidurias is influenced by the underlying defect (mut0, mut-, cblA, cblB). Pediatr Res. (2007) 62:225–30. 10.1203/PDR.0b013e3180a0325f 17597648

[8] de Baulny HO Benoist JF Rigal O Touati G Rabier D Saudubray JM Methylmalonic and propionic acidaemias: management and outcome. J Inherit Metab Dis. (2005) 28:415–23. 10.1007/s10545-005-7056-1 15868474

[9] Matsui SM Mahoney MJ Rosenberg LE. The natural history of the inherited methylmalonic acidemias. N Engl J Med. (1983) 308:857–61. 10.1056/NEJM198304143081501 6132336

[10] Cosson MA Benoist JF Touati G Dechaux M Royer N Grandin L. Long-term outcome in methylmalonic aciduria: a series of 30 French patients. Mol Genet Metab. (2009) 97:172–8. 10.1016/j.ymgme.2009.03.006 19375370

[11] Prada CE Al Jasmi F Kirk EP Hopp M Jones O Leslie ND. Cardiac disease in methylmalonic acidemia. J Pediatr. (2011) 159:862–4. 10.1016/j.jpeds.2011.06.005 21784454

[12] Traber G Baumgartner MR Schwarz U Pangalu A Donath MY Landau K. Subacute bilateral visual loss in methylmalonic acidemia. J Neuro Ophthalmol. (2011) 31:344–6. 10.1097/WNO.0b013e31822db480 21873889

[13] Leonard JV. The management and outcome of propionic and methylmalonic acidaemia. J Inherit Metab Dis. (1995) 18:430–4. 10.1007/BF00710054 7494401

[14] Goyens P Brasseur D Otte JB Marchau F De Laet CEC Liver transplantation for methylmalonyl CoA mutase deficiency. J Inherit Metab Dis. (1997) 20 (Suppl. 1):38.

[15] Morioka D Kasahara M Horikawa R Yokoyama S Fukuda A Nakagawa A. Efficacy of living donor liver transplantation for patients with methylmalonic acidemia. Am J Transplant. (2007) 7:2782–7. 10.1111/j.1600-6143.2007.01986.x 17908273

[16] Kamei K Ito S Shigeta T Sakamoto S Fukuda A Horikawa R. Preoperative dialysis for liver transplantation in methylmalonic acidemia. Ther Apheresis Dialysis. (2011) 15:488–92. 10.1111/j.1744-9987.2011.00974.x 21974703

[17] van't Hoff W McKiernan PJ Surtees RA Leonard JV Liver transplantation for methylmalonic acidaemia. Eur J Pediatr. (1999) 158 (Suppl. 2):S70–4. 10.1007/PL00014326 10603103

[18] Niemi A-K Kim IK Krueger CE Cowan TM Baugh N Farrell R Treatment of methylmalonic acidemia by liver or combined liver-kidney transplantation. J Pediatr. (2015) 166:1455–61.e1. 10.1016/j.jpeds.2015.01.051 25771389

[19] Chen PW Hwu WL Ho MC Lee NC Chien YH Ni YH. Stabilization of blood methylmalonic acid level in methylmalonic acidemia after liver transplantation. Pediatr Transplant. (2010) 14:337–41. 10.1111/j.1399-3046.2009.01227.x 19686300

[20] Sakamoto R Nakamura K Kido J Matsumoto S Mitsubuchi H Inomata Y. Improvement in the prognosis and development of patients with methylmalonic acidemia after living donor liver transplant. Pediatr Transplant. (2016) 20:1081–6. 10.1111/petr.12804 27670840

[21] Mc Guire PJ Lim-Melia E Diaz GA Raymond K Larkin A Wasserstein MP Combined liver-kidney transplant for the management of methylmalonic aciduria: a case report and review of the literature. Mol Genet Metab. (2008) 93:22–9. 10.1016/j.ymgme.2007.08.119 17964841 PMC2786260

[22] Nyhan WL Gargus JJ Boyle K Selby R Koch R. Progressive neurologic disability in methylmalonic acidemia despite transplantation of the liver. Eur J Pediatr. (2002) 161:377–9. 10.1007/s00431-002-0970-4 12111189

[23] Critelli K McKiernan P Vockley J Mazariegos G Squires RH Soltys K. Liver transplantation for propionic acidemia and methylmalonic acidemia: perioperative management and clinical outcomes. Liver Transplant. (2018) 24:1260–70. 10.1002/lt.25304 30080956

[24] Khanna A Gish R Winter SC Nyhan WL Barshop BA. Successful domino liver transplantation from a patient with methylmalonic acidemia. JIMD Rep. (2016) 25:87–94. 10.1007/8904_2015_480 26219882 PMC5059220

[25] Chakrapani A Sivakumar P McKiernan PJ Leonard JV. Metabolic stroke in methylmalonic acidemia five years after liver transplantation. J Pediatr. (2002) 140:261–3. 10.1067/mpd.2002.121698 11865284

[26] Chandler RJ Sloan J Fu H Tsai M Stabler S Allen R. Metabolic phenotype of methylmalonic acidemia in mice and humans: the role of skeletal muscle. BMC Med Genet. (2007) 8:64. 10.1186/1471-2350-8-64 17937813 PMC2140053

[27] Kaplan P Ficicioglu C Mazur AT Palmieri MJ Berry GT Liver transplantation is not curative for methylmalonic acidopathy caused by methylmalonyl-CoA mutase deficiency. Mol Genet Metabol. (2006) 88:322–6. 10.1016/j.ymgme.2006.04.003 16750411

[28] Spada M Calvo PL Brunati A Peruzzi L Dell'Olio D Romagnoli R. Early liver transplantation for neonatal-onset methylmalonic acidemia. Pediatrics. (2015) 136:e252–e6. 10.1542/peds.2015-0175 26077484

[29] Vernon HJ Sperati CJ King JD Poretti A Miller NR Sloan JL. A detailed analysis of methylmalonic acid kinetics during hemodialysis and after combined liver/kidney transplantation in a patient with mut 0 methylmalonic acidemia. J Inherit Metab Dis. (2014) 37:899–907. 10.1007/s10545-014-9730-7 24961826 PMC4373418

[30] Ma X Zhang Y Yang Y Liu X Yang Z Bao X. Epilepsy in children with methylmalonic acidemia: electroclinical features and prognosis. Brain Dev. (2011) 33:790–5. 10.1016/j.braindev.2011.06.001 21764232

[31] Kasahara M Sakamoto S Horikawa R Koji U Mizuta K Shinkai M. Living donor liver transplantation for pediatric patients with metabolic disorders: the Japanese multicenter registry. Pediatr Transplant. (2014) 18:6–15. 10.1111/petr.12196 24283623

[32] Andrews E Jansen R Crane AM Cholin S McDonnell D Ledley FD. Expression of recombinant human methylmalonyl-CoA mutase: in primary mut fibroblasts and *Saccharomyces cerevisiae*. Biochem Med Metab Biol. (1993) 50:135–44. 10.1006/bmmb.1993.1055 7903149

[33] Li M Dick A Montenovo M Horslen S Hansen R. Cost-effectiveness of liver transplantation in methylmalonic and propionic acidemias. Liver Transplant. (2015) 21:1208–18. 10.1002/lt.24173 25990417

[34] de Mello CF Begnini J Jimenez-Bernal RE Rubin MA de Bastiani J da Costa E Jr Intrastriatal methylmalonic acid administration induces rotational behavior and convulsions through glutamatergic mechanisms. Brain Res. (1996) 721:120–5. 10.1016/0006-8993(96)00117-5 8793091

[35] Smith CB Sokoloff L The energy metabolism of the brain. In: Davidson AN Thompson RHS, editors. The Molecular Basis of Neuropathology. London: Edward Arnold Limited (1981).

[36] Hauser NS Manoli I Graf JC Sloan J Venditti CP. Variable dietary management of methylmalonic acidemia: metabolic and energetic correlations. Am J Clin Nutr. (2011) 93:47–56. 10.3945/ajcn.110.004341 21048060 PMC3001598

[37] Manoli I Sysol JR Li L Houillier P Garone C Wang C. Targeting proximal tubule mitochondrial dysfunction attenuates the renal disease of methylmalonic acidemia. Proc Natl Acad Sci USA. (2013) 110:13552–7. 10.1073/pnas.1302764110 23898205 PMC3746875

[38] Kruszka PS Manoli I Sloan JL Kopp JB Venditti CP. Renal growth in isolated methylmalonic acidemia. Genet Med. (2013) 15:990–6. 10.1038/gim.2013.42 23639900 PMC4149057

[39] Fujisawa D Nakamura K Mitsubuchi H Ohura T Shigematsu Y Yorifuji T. Clinical features and management of organic acidemias in Japan. J Hum Genet. (2013) 58:769–74. 10.1038/jhg.2013.97 24067294

[40] Morath MA Horster F Sauer SW. Renal dysfunction in methylmalonic acidurias: review for the pediatric nephrologist. Pediatr Nephrol. (2013) 28:227–35. 10.1007/s00467-012-2245-2 22814947

[41] Israeli M Klein T Sredni B Avitzur Y Mor E Bar-Nathen N. ImmuKnow: a new parameter in immune monitoring of pediatric liver transplantation recipients. Liver Transplant. (2008) 14:893–8. 10.1002/lt.21426 18508374

[42] Sloan JL Manoli I Venditti CP. Liver or combined liver-kidney transplantation for patients with isolated methylmalonic acidemia: who and when? J Pediatr. (2015) 166:1346–50. 10.1016/j.jpeds.2015.03.026 25882873

[43] Inker LA Schmid CH Tighiouart H Eckfeldt JH Feldman HI Greene T. Estimating glomerular filtration rate from serum creatinine and cystatin C. N Engl J Med. (2012) 367:20–9. 10.1056/NEJMoa1114248 22762315 PMC4398023

