# Corrigendum: The Value of Liver Transplantation for Methylmalonic Acidemia

**DOI:** 10.3389/fped.2020.00126

**Published:** 2020-04-02

**Authors:** Yi-Zhou Jiang, Li-Ying Sun

**Affiliations:** ^1^Intensive Care Unit, Beijing Friendship Hospital, Capital Medical University, Beijing, China; ^2^Liver Transplantation Center, Clinical Center for Pediatric Liver Transplantation, National Clinical Research Center for Digestive Diseases, Beijing Friendship Hospital, Capital Medical University, Beijing, China; ^3^Beijing Key Laboratory of Tolerance Induction and Organ Protection in Transplantation, Beijing, China

**Keywords:** methylmalonic acidemia, methylmalonic acid, liver transplantation, metabolic, decompensation

In the original article, there was a mistake regarding references in Table 1 as published. The corrected Table 1 appears below.

**Table 1:** Outcomes of LT/CKLT for patients with MMA.

**References**	**Age at Tx**	**Procedure**	**Follow-up**	**Metabolic decompensation/crisis time**		**MMA level (P/CSF: nmol/mL U:** **μmol/mmol Cr)**		**Dietary protein (g/kg/d)**		**Neurological damage/ DQ**		**Renal dysfunction (eGFR:mL/min/1.73 m**^****2****^		**Developmental delay/SD of height**	
				**Pre**	**Post**	**Pre**	**Post**	**Pre**	**Post**	**Pre**	**Post**	**Pre**	**Post**	**Pre**	**Post**
Kaplan et al. (27)	19 m	OLT	10 y	Y	Y (only twice)	P:574 ± 431	P:220 ± 79	1.7	NA	Increased subarachnoid space	Acute lesion in right globus pallidus, then resolved^&^	N	eGFR = 77	Between the 25th and 50th percentiles	−2SD
						U:9307 ± 4923	U:3656 ± 2271								
						CSF: 1103	CSF:901 ± 263								
Mc Guire et al. (21)	5 y	CKLT (OLT)	10 m	Y	N	P:20–2591	P:25–525	1.95	NA	Y (cerebellar stroke)	Y	Y	N	Failure to thrive	NA
						U:1101–13962	U:116–1895								
Chen et al. (19)	0.9–2.1 y	LDLT (*n* = 4)	0.2–7.7 y	2.73/y	0.08/y	P:87.5–204	P:63.2–87	0.66–1.00	1.37–2.80	NA	NA	N	N	Development all continued	
Morioka et al. (15) Kamei et al. (16)	7–90 m	LDLT (*n* = 7)	19–53 m	Y	N	P:268.0	P:99.4	1.0	3.0	The global cognitive index of the McCarthy scale and the Denver development quotient were improved but did not reach normal values		N	N	−2	−2
						P:47.0	P:59.2	1.2	2.5			N	N	−2	−2
						P:143.0	P:36.4	0.7	2.5			N	N	−3.14	−2
						P:39.0	P:29.3	2.0	3.0			N	N	−2	−1
						P:375.0	P:87.8	1.0	2.5			N	N	−1.3	−0.6
						P:1970.0	P:232.0	–^#^	–			Y	–	–	–
						P:166.0	P:13.8	1.5	2.5			N	N	−3	−2
		LDLT (*n* = 3)				P:278.0	P:59.6	NA	NA	NA	NA	N	N	NA	NA
						P:702.0	P:124.4								
						P:255.0	P:8.5								
Vernon et al. (29)	28 y	CKLT	18 m	Y	N	P: 6965 ± 1638	P:234 ± 100	Restricted	Not restricted	Optic neuropathy, leukoencephalopathy	Stable visual function, tremor persists	Y	N	Worsening generalized debility	Able to walk
Spada et al. (28)	3 y	Whole LT	12 y	Y	N	P: sustained (~80%) and stable reduction	0.8	1.5	Normal intellectual development	N	Y	NA	NA		
	9 m	Split-LT	2 y	Y	N	P:124.4	P:43.5	0.8	1.8	Adequate neurologic development		N	N	NA	NA
Niemi et al. (18)	Mean 8.2 y (0.8–20.7)	LT^*^ (*n* = 6) CKLT (*n* = 8)	Mean 3.25 ± 4.2 y	Y	N	P:1648 ± 1492	P:305 ± 108	1.6 (Natural protein 0.3–1.9)	1.6 (Natural protein 0.6–1.8)	Maintained neurodevelopmental abilities	Y (*n* = 8)	N	Present in 12 patients (86%)	Maintained or improved	
Khanna et al. (24)	28 y	OLT (domino donor)	11 m	Y	N	P:445.9 ± 257.0	P:333.3 ± 117.7	Y	1.0–1.9 (liberalized)	Increasing neurologic disability	NA	>60	51.0 ± 12.1^†^	Altered gait, and slower speech	NA
						U:5277 ± 1968	U:1068 ± 384								
Sakamoto et al. (20)	7 y	LDLT (*n* = 13)	4–16 y (mean 8.1 y)	0	0	P: ~75–240 (mean)	P: ~5–170 (mean)	1.2	Less	41	53	N	N	−2.0	−2.0
	5 y			3	0			0.7	Less	43	48	N	N	−3.1	−2.0
	1 y			3	0			1.5	1.65–1.8	49	54	N	N	−3.0	−2.0
	8 m			1	0			1.2	1.3	NA	32	N	N	−2.8	−0.2
	11 m			3	1			0.9	1.5	55	48	N	N	−1.4	−1.8
	5 y			5	5			1.7	0.95	NA	23	N	N	−4.3	−4.4
	10 m			2	0			1.5	1.0–1.5	63	55	N	N	−2.5	−1.3
	12 m			2	0			0.7	1.0–1.5	57	42	N	N	−2.5	−1.7
	9 m			3	2			1.3	1.0	NA	NA	N	N	−3.2	−0.6
	8 m			1	0			1.3	1.2	NA	NA	N	N	1.5	0.8
	2 y			3	0			1.0	1.0–1.5	60	54	Y	Y^※^	−3.6	−1.9
	2 y			5	1			2.0	1.0–1.5	NA	NA	N	N	−3.6	−3.2
	10 m			1	0			1.5	Not restricted	70	NA	N	N	−0.7	0.0
Critelli et al. (23)	6.6 y	Kidney/split liver	3.1 y	Y	N	P: 745 (mean)	P: 154.9 (mean)	1.6–2.0	1	NA	NA	56	78	Mild	NA
	21.6 y	CKLT	1.6 y	Y	N			1.45–1.75	1.0–1.1			40	70	Extremely low to borderline	
	7.4 y	CKLT	4.1 y	Y	N			1.6–2.0	1.43			66.2	142	Moderate to severe	
	15.5 y	CKLT	11.6 y	Y	N			1.3	0.76–0.95			40	68^§^	Mild	
	9.4 y	CKLT	3.6 y	Y	N			0.98–1.18	1.3–1.5			65	88	No formal testing	
	1.9 y	OLT	1 y	Y	N			0.83	1.0–1.2			96.8	128	Borderline	

*NA, not available; OLT, orthotopic liver transplantation; LDLT, living donor liver transplantation*.

& *72 days post-transplantation, MRI with diffusion-weighted imaging of brain demonstrated an acute lesion in the right globus pallidus but has never manifested clinical signs of extrapyramidal tract disease. Subsequent MRI 18 months later showed resolution of the basal ganglion lesion*.

# *Died of sepsis on postoperative day 44*.

* *One underwent liver retransplantation because of hepatic artery thrombosis*.

† *The postoperative period was complicated by acute kidney injury. The renal function improved progressively*.

※ *Acute renal failure occurred after using contrast medium for endoscopic retrograde cholangiopancreatography*.

§ *Underwent a renal biopsy 17 months after CLKT, which showed mild tubulointerstitial injury*.

The authors apologize for this error and state that this does not change the scientific conclusions of the article in any way. The original article has been updated.

